# Dataset of plasma and aqueous humor cytokine profiles in patients with exudative age related macular degeneration and polypoidal choroidal vasculopathy

**DOI:** 10.1016/j.dib.2018.05.085

**Published:** 2018-05-24

**Authors:** Praveen Kumar Balne, Rupesh Agrawal, Veonice Bijin AU, Bernett Lee, Arkasubhra Ghosh, Swaminathan Sethu, Mukesh Agrawal, Raja Narayanan, John Connolly

**Affiliations:** a National Healthcare Group Eye Institute, Tan Tock Seng Hospital, 11 Jalan Tan Tock Seng, Singapore 308433, Singapore; b Singapore Eye Research Institute, Singapore 169856, Singapore; c Institute of Molecular and Cell Biology, Singapore 138673, Singapore; d Singapore Immunology Network, Singapore 138648, Singapore; e GROW Research Laboratory, Narayana Nethralaya Foundation, 560099, India; f Vimta Labs Limited, Hyderabad, Telangana 500051, India; g L. V. Prasad Eye Institute, Hyderabad, Telangana 500034, India

**Keywords:** Exudative age related macular degeneration, Choroidal neovascularization, Polypoidal choroidal vasculopathy, Cytokine profiles, Aqueous humor, Plasma

## Abstract

In this report the data was obtained from a prospective case-control study with a sample size of sixteen patients with exudative age related macular degeneration (AMD) due to choroidal neovascularization (CNV) and eighteen patients with polypoidal choroidal vasculopathy (PCV) and fifty controls (cataract patients without any other ocular diseases). Luminex bead based multiplex assay with a panel of 41 analytes was used to study the cytokine levels in plasma and aqueous humor.

**Specifications Table**

**Table:** 

Subject area	*Biology*
More specific subject area	*Plasma and aqueous humor cytokine levels in exudative age related macular degeneration (AMD)*
Type of data	*Table*
How data was acquired	*Bead based multiplex assay using the Luminex xMAP technology*
Data format	*Raw*
Experimental factors	*Plasma and aqueous humor samples were collected from study subjects and each sample was analyzed for 41 cytokine levels*
Experimental features	*Milliplex*® *MAP human cytokine / chemokine magnetic bead panel -1 kit (Millipore, USA) with FlexMAP 3D (Luminex*®*) platform was used for cytokine profiling in each sample*
Data source location	*Singapore*
Data accessibility	*Data is with this article*

**Value of the data**

• Here we report the plasma and aqueous humor cytokine levels in patients with exudative AMD, PCV and controls (cataract patients without any other ocular diseases).
• In exudative AMD patients, aqueous humor cytokines GRO, MDC and MIP-1α were significantly higher than controls (*p* < 0.04).
• In PCV patients, aqueous humor cytokines GRO, MDC, MIP-1α, MIP-1β, IL-8, IP-10 and MCP levels were significantly higher than controls (*p* < 0.03).
• No significant differences were seen in plasma cytokine levels between cases and controls.
• None of the plasma and aqueous humor cytokines showed significant differences between exudative AMD and PCV.
• This data will help researchers to understand the differences in cytokine expression levels between patients with Exudative AMD, PCV and controls.
• This data will also help researchers to understand the differences between plasma and aqueous humor in patients with Exudative AMD, PCV and healthy controls.

## 1 Data

The data presented in this article was obtained from a prospective case-control study and shows the concentration of 41 cytokines in plasma and aqueous humor from patients with exudative age related macular degeneration (AMD), polypoidal choroidal vasculopathy (PCV) and healthy controls (Table 1).

## 2 Experimental design, materials and methods

In a prospective case-control study, sixteen patients who were diagnosed with exudative age related macular degeneration (AMD) due to choroidal neovascularization (CNV) and eighteen patients with polypoidal choroidal vasculopathy (PCV) and fifty age and gender matched cataract patients without any other ocular complications (controls) were recruited. The study was approved by the Institutional review board of Tan Tock Seng Hospital, Singapore and adhered to the tenets of the Declaration of Helsinki. Prior informed consent was obtained from all the subjects. Five milliliters of peripheral venous blood collected aseptically from 14 AMD patients, 18 PCV patients and fifty controls and approximately 200 µL of aqueous humor collected from 16 AMD patients, 16 PCV patients and 41 controls using standard protocols were used for cytokine profiling [1]. Clinical samples were transferred onto the ice immediately after the collection and stored at −80 °C freezer until cytokine analysis.

Just before analysis clinical samples were thawed on ice and centrifuged for 5 minutes at 3000 rpm. Supernatant and plasma were separated from aqueous humor and plasma, respectively and transferred onto the ice. Twenty five microliters of each sample was analyzed for a panel of 41 cytokines (Table 1) by Luminex bead based multiplex assay using Milliplex® MAP human cytokine / chemokine magnetic bead panel -1 kit (Millipore, USA) with FlexMAP 3D (Luminex®) platform following the manufacturer׳s instructions [1], [2]. Plasma and aqueous humor cytokine concentrations (pg/mL) were mentioned in Table 1 and mean±SD concentrations in each group were mentioned in Table 2.

**Table 1:** Mean±SD concentrations (pg/mL) of plasma and aqueous humor cytokine concentrations in both cases and controls. Note: Empty cells represent samples negative for that particular analytes.

	**Plasma controls**		**Plasma AMD**		**Plasma PCV**		**Aqueous controls**		**Aqueous AMD**		**Aqueous PCV**	
	**Mean**	**SD**	**Mean**	**SD**	**Mean**	**SD**	**Mean**	**SD**	**Mean**	**SD**	**Mean**	**SD**
**EGF**	34.37	44.86	29.7	35.86	19.29	12.21	2.29	0.78	2.7	0.98	2.32	0.93
**Eotaxin**	94.81	53.9	116.2	44.69	102.59	40.73	7.44	3.07	9.44	2.49	8.42	5.37
**FGF2**	133.04	140.92	96.23	69.19	131.11	105.96	17.76	10.45	23.94	11.85	16.71	11.38
**Flt3l**	123.58	163.91	55.36	36.45	75.16	82.94	6.38	3.15	7.17	4.05	6.58	5.12
**Fractalkine**	109.64	116.54	71.73	40.48	87.26	53.8	37.62	17.66	44.87	21.32	48.1	24.36
**G-CSF**	106.67	159.63	212.41	442.16	90.74	67.08	2.88	1.78	5.96	4.28	8.75	10.64
**GM-CSF**	42.99	146.47	49.37	114.28	24.08	22.51	1.57	0.63	1.69	0.58	1.91	0.96
**GRO**	153.64	103.76	143.85	249.06	124.03	74.77	28.92	11.65	43.87	15.7	61.45	69.35
**IFN-a2**	89.99	227.64	20.04	6.13	41.04	50.9	6.28	2.95	6.17	3.81	8.56	4.29
**IFN-g**	55.83	76.95	43.61	51.96	38.23	33.93	0.66	0.5	0.5	0.37	0.49	0.27
**IL10**	5.14	11.05	3.12	2.28	15.04	30.15	1.04	0.73	2.4	3.84	4.03	7.4
**IL12p40**	50.25	131.09	15.47	17.81	34.23	55.93	3.4	2.8	2.96	1.87	3.8	3.65
**IL12p70**	25.16	52.73	13.69	13.57	31.89	48.17	1.44	0.71	1.09	0.37	0.87	0.84
**IL13**	17.42	24.21	31.19	56.86	17.01	20.98	0.63	0.77	1.27	1.63	1.52	1.47
**IL15**	2.91	4.04	1.29	1.34	4.26	8.76	2.65	0.75	2.94	1.3	2.86	1.19
**IL17a**	35.88	47.08	19.9	34.93	22.33	19.8	0.85	0.86	1.32	0.63	2.05	0.44
**IL1a**	143.41	241.42	131.65	228.37	56.57	63.77	0.47	0.29			0.98	0.81
**IL1b**	4.7	13.15	1.42	1.85	6.99	18.91	0.4	0.28	0.28	0.12	0.46	0.18
**IL1ra**	28.95	36.89	36.95	85.89	41.98	44.84	5.06	7.11	8.6	12.39	16.54	17.68
**IL2**	15.3	28.69	6.55	7.34	11.38	15.32	0.64	0.51	0.59	0.3	0.6	0.35
**IL3**	0.53	0.53			0.31	0.2						
**IL4**	12.26	15.08	20.78	15.72	19.75	25.3	2.85	2.48	0.12			
**IL5**	2.92	10.21	4.24	7.57	6.81	17.55	0.31	0.34	0.28	0.29	0.34	0.4
**IL6**	12.64	17.98	5.35	6.19	8.34	12.78	3.29	9.17	8.64	13.25	8.44	13.59
**IL7**	8	15.55	3.08	2.85	4.27	6.5	1.96	1.97	1.26	0.83	1.58	0.59
**IL8**	15.42	16.17	10.67	12.45	10.55	7.14	8.47	6.11	13.46	10.61	236.64	799.78
**IL9**	2.85	4.96	1.58	1.62	29.7	75.4	0.55	0.31	0.32	0.11	0.29	0.18
**IP10**	327.31	517.44	361.31	394.37	285.33	233.57	257.23	184.76	338.47	233.22	2135.08	5330.65
**MCP-1**	373.31	101.07	384.24	113.97	365.17	127.88	1098.99	465.34	1253.65	394.01	3619.71	4721.22
**MCP-3**	21.13	19.15	37.37	64.73	27.09	21.05	8.27	3.82	9.52	6.72	19.6	22.42
**MDC**	535.96	256.71	587.55	216.45	485.84	148.26	18.75	11.21	36.83	26.67	44.39	28.3
**MIP-1a**	11.4	12.18	10.25	13.1	9.32	8.73	6.25	3.58	13.02	5.85	12.31	7.81
**MIP-1b**	53.77	61.35	51.24	49.45	53.42	56.5	10.86	6.63	19.15	21	15.85	6.87
**PDGF-aa**	327.68	327.11	192.47	304.05	216.79	168.48	30.11	20.81	33.59	19.33	45.3	28.8
**PDGF-ab/bb**	377.63	681.41	764.6	1837.83	243.42	162.78	6.47	4.55	4.74	4.26	6.66	2.64
**RANTES**	711.33	554.86	680.21	997.16	625.8	381.75	4.06	5.06	3.44	4.05	1.83	0.51
**Scd40l**	1094.54	574.32	421.17	379.18	951.31	732.54	1.39	1.95	1.57	3.11	2.5	3.32
**TGF-a**	4.82	7.36	9.24	11.71	2.79	3.08					0.47	0.37
**TNF-a**	10.1	8.35	9.75	4.13	9.49	4.16	0.32	0.13	0.3	0.2	0.4	0.19
**TNF-b**	76.2	182.81	93.82	208.04	57.81	73.79	0.78	0.81	0.52	0.1		
**VEGF**	963.02	1478.15	688.77	845.02	506.81	471.15	55.85	34.73	37.38	20.46	50.22	24.22

## Appendix A Supplementary material

Supplementary material **Table 1:** Concentrations of 41 cytokines (pg/mL) in plasma and aqueous humor of patients with exudative age related macular degeneration due to choroidal neovascularization, polypoidal choroidal vasculopathy and age and gender matched controls. Note: Empty cells represent samples negative for that particular analytes.
